# Agenda for specialty section in addiction medicine

**DOI:** 10.4103/0019-5545.44741

**Published:** 2008

**Authors:** T. S. Sathyanarayana Rao, M. N. Anil Kumar

**Affiliations:** Department of Psychiatry, JSS University, JSS Medical College Hospital, Mysore - 57 0004, India; 1 Department of Psychiatry, K S Hedge Medical Academy, Mangalore, India

## INTRODUCTION

Addictive behavior is any activity or behavior that has become the major focus of a person's life to the exclusion of other activities, or that has begun to harm the individual or others physically, mentally, or socially. Addictive behavior increases the risk of disease and/or associated personal and social problems. They are often experienced subjectively as “loss of control”, that is, the behavior continues to occur despite volitional attempts to abstain or moderate use. Addiction implies psychological dependence, physical dependence, and a set of withdrawal symptoms if the substance is unavailable.[1] There are many common characteristics among the various addictive behaviors. First, the individual becomes obsessed (constantly thinks) of the object, activity, or substance and will seek it out, often to the detriment of work or interpersonal relationships. The person will compulsively engage in the activity even if he does not want to. Upon cessation of the activity, withdrawal symptoms of irritability, craving, and restlessness will often occur. The person does not appear to have control as to when, how long, or how much he will continue the behavior. He often denies problems resulting from his engagement in the behavior even though others can see the negative effects of it. Further, individuals with addictive behaviors will usually have low self esteem.[2]

## BURDEN OF PROBLEM

Substance-related disorders have become matters of global concern because of impact on individual health, familial and social consequences, criminal and legal problems, and the effects on national productivity and economy. All the problems from use to dependence and from the most innocuous substance like tea/coffee to the most hard substances, contribute to the cost that the human society or a particular nation has to bear. In India, although alcohol use in ancient times and cannabis/affim (opium) in more recent times have been known and reported for some time, substance use problems have been recognized to have a significant importance as a public health problem only very recently. They are of particular concern among slum dwellers, transport workers, and youth.[3]

Tobacco is the substance most easily accessible worldwide as well as in India. About one-fifth of the Indian population abuse tobacco, about 70% of this in the smoking form.[4] Passive smoking is a significant health hazard. There are 700,000 deaths per year due to smoking and 800,000–900,000 deaths per year due to all forms of tobacco use in India. Many of the deaths (>50%) are occurring below 70 years.[5]

An estimated 34–42% of adult Indian population reports having used alcohol in their lifetime; 5–7% has been estimated to be abuser of alcohol; and 10–20 million persons have been estimated to be in need of treatment for dependence. A survey in India revealed that medical professionals had a higher rate of use and heavy use than general population.[5] Nearly 20–30% of admissions and consultations are due to alcohol-related problems in different healthcare settings but are under-recognized by primary care physicians.[6]

With respect to heroin, India has now changed from being a transit country to a user country. General population surveys in India show opioid abuse rate as high as 24.8%. Another great concern especially with injectable opioids is spread of AIDS. Further, cannabis abuse in general population in India is 33% and abuse of tranquilizers is as high as 54%.[3] Remediation at central level:

‘The Drug Deaddiction Program’ was started in 1987–88 with the establishment of five deaddiction centers in central institutions, viz. AIIMS, New Delhi; Dr. RML Hospital, New Delhi; Lady Harding Medical College and Hospital; New Delhi; JIPMER, Pondicherry; and PGI, Chandigarh. The center at NIMHANS, Bangalore was established later as the sixth center. More recently, in 1999, a separate deaddiction center with inpatient setup was started in Central Institute of Psychiatry, Ranchi. The Ministry of Health and Family Welfare is mainly involved in providing treatment services to the addicts, whereas the Ministry of Social Justice and Empowerment deals with other aspects of the problem like awareness creation, counseling, and rehabilitation. From 1992–93, the scope of the scheme was enlarged to include assistance to State Governments/Union Territories for developing deaddiction centers in identified medical colleges/district-level hospitals.

## WHAT WORKS IN SUBSTANCE-USE DISORDERS?

Treatment of substance-use disorders typically involves a combination of pharmacotherapy and psychosocial interventions. Techniques for medically assisted detoxification are widespread and effective. The following are the different strategies that are found useful in management of substance dependence.[7]

### i) Home versus inpatient detoxification

Published reports have consistently failed to find any difference in outcome between long and short inpatient detoxification programs. Clients can usually complete home detoxification in 5–9 days. In ideal circumstances they are visited twice daily for the first three days and medication is supervised by a relative. Clients are breathalyzed and medication withheld if they have consumed significant amounts of alcohol.

### ii) Brief interventions

Brief interventions are short, focused discussions (often of less than 15 minutes) that can reduce alcohol consumption in some individuals with hazardous drinking. They are designed to promote awareness of the negative effects of drinking and to motivate change. Brief interventions were shown to be moderately effective in the nontreatment-seeking groups, especially for those with less-severe alcohol problems.

### iii) Abstinence versus controlled use

This is a great debate. The goal of controlled (moderate or nonproblem) drinking usually includes some limit on alcohol consumption (e.g., 4 units/day) provided that drinking does not lead to signs of dependence, intoxication, or social, legal and health problems. This runs contrary to the abstinence-based philosophy of Alcoholics Anonymous. Controlled drinking may be an option for young, socially stable drinkers with short, less-severe drinking histories. An individual's belief that controlled drinking is an achievable goal is also a good prognostic factor. Most authors agree that controlled drinking should not be recommended for people with heavy dependence. Controlled drinking is an attractive option for public health strategies aimed at nondependent problem drinking. This principle can also be tried for other substances.

### iv) Alcoholic Anonymous

Based on Minnesota model, they involve the recognition that alcoholism is a relapsing illness that requires complete abstinence. Clients acknowledge the problems due to their alcoholism and are encouraged to attend 90 meetings in 90 days. Participants may engage the support of a sponsor who is an AA member who has been sober for at least one year. Similarly, there is narcotic anonymous for opioid addicts and nicotine anonymous for nicotine dependents. The evidence suggests that this twelve-step approach is at least as effective as most structured psychotherapies but not superior to any alternative treatments.

### v) Psychological therapies

- Motivational enhancement therapy
- Cognitive behavioral therapy
- Social skills training
- Community reinforcement approach
- Social behavior and network therapy
- Contingency management
- Cue exposure

### vi) Therapeutic community

It requires prolonged residence (often 12–18 months) and it is extremely expensive. Most studies of therapeutic communities are conducted without control groups and the lack of randomization probably leads to selection bias in favor of more motivated patients.

### vii) Drug treatments

Disulfiram, naltrexone, and acamprosate are currently the only treatments approved for the management of alcohol dependence. Other drugs which have been used for the same are topiramate, citrated calcium carbimide, SSRIs, tiapiride, nalmefene, metronidazole, and ondansetron. The oldest among these is disulfiram, being used from 1950s. Naltrexone was launched in India in 1998 and acamprosate in 2002. However, none has been proved to be significantly better than the other agents. In other words, all are equally effective.[5]

Bupropion has been said to be the first line drug for nicotine craving. Another first line agent is nicotine itself, in the form of gum, inhaler, lozenge, patch, and nasal spray. Clonidine and nortriptyline are said to be the second line drugs. But the success rate with each of these drugs is marginal.[8]

Sustained release bupropion is also considered to be effective as anticraving agent in cannabis dependence. Nefazodone also significantly reduces cannabis withdrawal related anxiety and muscle pain, while having no effect on dysphoria. The embryonic work on CB1 selective cannabinoid receptor antagonists such as SR141716 (rimonabant) has demonstrated that it successfully blocks the acute psychological and physiological effects of smoked cannabis in experienced human consumers and is well tolerated. There has been even less work on the use of cannabis agonists. However, this is a promising area for future research as a number of oral and inhaled THC preparations are being developed for medical cannabis trials.[9]

Maintenance treatment is increasingly emphasized as the main therapy of opioid dependency. Abstinence-oriented therapy is nevertheless an important alternative, mainly psychological, and has considerable advantages for those benefitting from this strategy. Methadone or buprenorphine in tapering doses are used for detoxification along with α2 adrenergic agonists like clonidine or lofexidine. Time-limited agonist therapy is a specific approach using agonist stabilization as the basis for therapeutic interventions preceding tapering off of the maintenance therapy. The preventive effect of naltrexone and other antagonists is well known but the attrition rate is very high. It is also feasible for addicts to be introduced to naltrexone by detoxification under anesthesia or sedation.[10]

## ADDICTION MEDICINE AS A SPECIALTY

As elaborated above, substance use is affecting a disproportionately large section of people of India. The prevalence is more than that of all severe mental disorders combined. When it comes to health problems, it should not be gauged merely by its prevalence, but by the health burden and social cost that it wreaks on the society. Psychiatric comorbidities like mood, anxiety, and psychotic disorders are also very commonly associated with substance dependence. Thus drug addiction is a titanic problem that needs special clinical attention.

Today we are equipped with an arena of treatment strategies, both pharmacological and nonpharmacological. What we need is just an improvisation in its implementation. Treatment can be better systematized when addiction medicine is upgraded as a specialty. The specialty section on addiction medicine needs to fulfill the following aims and objectives.[6]

1. Promote and advance the bio-psycho-social contributions to the science and practice of addiction medicine in all its different manifestations.
2. Promote the improvement of the health of persons directly or indirectly impacted by substance-related problems.
3. Promote prevention, control, treatment, and relief of all substance-related problems, as well as conditions which constitute high risk for potential drug-related problems. This involves encouraging and enabling the members of the society to explore and pursue ‘evidence based’ treatment strategies and prevention methods. This includes liaison with other stake holders, including but not limited to government agencies overseeing health, social justice and welfare, and finance and human development; nongovernmental agencies involved in direct care of persons with substance abuse.
4. Promote other medical specialties and primary healthcare providers. This also requires sharing the expertise of members of the society and other professionals involved in diverse fields such as school mental health, community based rehabilitation, epidemiology, drug trials, maintaining disease registries, to mention a few.
5. Actively engage in advocacy in order to influence policy making and legislation, keeping in mind that substance-use disorders and their consequences constitute a grave public health disorder which imposes an unacceptably severe burden on the health and well being of the people of India.
6. Formulate and recommend standards of treatment and rehabilitation of persons or populations affected directly or indirectly by substance-use disorders. In addition, it will be necessary to obtain recommendations for syllabi and commission resources for the education and training of medical and nonmedical personnel involved in the field.
7. Safeguard the interest of psychiatrists and fellow professionals engaged in the practice of addiction medicine in India.
8. Promote research in the field of addiction science with a focus on translating the benefits of research to the bedside and propagate the use of evidence-based interventions in the daily delivery of mental healthcare.

## FUNCTIONING OF ADDICTION MEDICINE AS A SPECIALTY SECTION

The following are some of the activities that are being suggested as functions of specialty section of addiction medicine:[6]

- A web portal, linked to the web page of the Indian Psychiatric Society, which will initially host information of relevance to the practice of addiction medicine in India, with direct and indirect links to published or unpublished scientific papers, databases, treatment protocols, available treatment agencies, online resources, etc; and later e-learning sites for online education and training.
- Coordinating Continuing Medical Education (CME) pertaining to the aspect of the science and practice of addiction medicine, with special emphasis on underserved areas like the northeastern states, Andaman and Nicobar Islands, etc.
- Enabling and commissioning position papers on the impact of substance abuse in India, with a view to influence public policy.
- Engaging with relevant organizations to promote demand-reduction strategies, such as the police to prevent drinking and driving, primary health providers, and emergency rooms for early detection and brief intervention
- Pursuing the aim of working to safeguard vulnerable populations, for example, inoculating children and adolescents through skills training and sensitizing physicians and pharmacists to prevent over the counter drug abuse.
- Examining the possibility of creating a national registry, using standardized data-entry protocols.

## CONCLUSION

Substance-use disorders form more than 20% of the case load of psychiatric practitioners. However, practitioners have an attitude of therapeutic nihilism toward the addicted patient. This may be due to various reasons like lack of education about alcohol and substance abuse, attitude of hostility or aversion toward alcoholics, inability or discomfort to deal with the associated psychosocial issues, and learned pessimism about treatment – especially based on experience treating late stage alcoholics. However for effective outcome, early identification and treatment should be the goal. This is possible with more sophistication in the science of addiction medicine. Thus, evolving of a specialty section of addiction medicine is the need of the day.
